# Constructing a measure for self-perceived open organizational culture in a university hospital pharmacy

**DOI:** 10.3389/fmed.2024.1428941

**Published:** 2024-10-16

**Authors:** Wim J. R. Rietdijk, Madzy Maljaars-Hendrikse, Monique van Dijk, Romana F. Malik, Ngoc Tan, P. Hugo M. van der Kuy

**Affiliations:** ^1^Department of Clinical Pharmacy, Erasmus MC, Rotterdam, Netherlands; ^2^Legal Department, Erasmus MC, Rotterdam, Netherlands; ^3^Section Nursing Science, Department of Internal Medicine, Erasmus MC, Rotterdam, Netherlands; ^4^Department of Obstetrics and Gynaecology, Amsterdam University Medical Center, Amsterdam, Netherlands

**Keywords:** open organizational culture, healthcare, validation, survey, reliability, construct validation

## Abstract

**Background:**

An open organizational culture in the workplace represents an environment where information, ideas, and feedback are freely exchanged among all members, regardless of position or rank. Currently, there are no valid survey instruments to measure this culture within a healthcare context. To address this gap, we developed a survey instrument to measure self-perceived open organizational culture at a university pharmacy using a test re-test study design.

**Methods:**

Data were collected during classroom training on basic mediation skills study. Participants completed the same questionnaire before (test phase) and after the training (validation phase). The questionnaire included statements assessing open organizational culture. The data were analyzed using standardized psychometric methods, including correlations, Exploratory Factor Analysis (structural validity), and construct validity by correlating the open organizational culture scores with the Interpersonal Communication Inventory.

**Results:**

In the test phase, 191 participants (161 females, 84%; response rate = 39.7%) contributed to the initial construction of the self-perceived open organizational culture survey instrument. In the validation phase, 81 of the original respondents completed the questionnaire again. Three latent factors were identified, retaining 22 of the 37 items: “enabling systems” (7 items), “open behavior” (8 items), and “trusting and supporting coworkers” (7 items). High correlations were found among the three factors (*r* > 0.6), and between these factors and the Interpersonal Communication Inventory (*r* > 0.35). Cronbach’s alphas were all above 0.85, indicating good internal consistency. During the validation phase, the factors demonstrated high internal consistency, test/re-test correlations, and agreement.

**Conclusion:**

This study presents a 22-item survey instrument for measuring individual differences in self-perceived open organizational culture within a university hospital pharmacy. The instrument demonstrates internal consistency and construct validity. Further validation of its psychometric properties and testing in other healthcare departments are recommended.

## Introduction

Organizational culture is crucial in understanding and addressing workplace challenges (1, 2). It encompasses shared beliefs, norms, and values that shape employee behavior, attitudes, and well-being. Additionally, organizational culture reflects an organization’s processes, practices, and activities, influencing employee performance (3–5). In healthcare, recent studies on organizational processes indicate a growing emphasis on fostering an “open” culture (4, 5).

An open organizational culture (OOC) in healthcare involves an environment where information, ideas, and feedback are freely exchanged among all members, regardless of rank and position (4–8). High-performing healthcare teams consistently exhibit an OOC, where open communication fosters creativity and drives significant breakthroughs (8). This culture promotes trust and mutual respect, empowering individuals to voice their thoughts and contribute to shared goals (9, 10). In healthcare, OOC elements overlap with general organizational psychology but also include specific aspects such as patient orientation and psychological safety (5).

The relevance of an OOC in healthcare is heightened by current challenges, such as workplace safety concerns and the increasing demand for transparent professional communication (5, 11). This is especially critical given the current of personnel shortages and mounting pressure on healthcare systems due to aging populations (4–6, 12), which can increase the likelihood of workplace conflicts among colleagues increases. Implementing and monitoring an OOC can help address these issues effectively.

From both research and management perspectives, it is essential to objectively measure and monitor the perceived OOC of employees within healthcare departments. However, to our knowledge, no valid and reliable survey instrument currently exists in the literature that can measures this. An earlier Delphi study identified key aspects of an OOC specific to healthcare departments (5). Building on these findings, we used them as a basis for developing a survey instrument to measure self-perceived OOC. This procedure aligns with the Checklist for Reporting of Survey Studies (CROSS) guidelines for survey measurement research (13).

To achieve this, we used data from a study on the effect of a classroom training on mediation skills to assess the test–retest reliability, structural and construct validity, and internal consistency of a newly developed questionnaire on self-perceived OOC at a university hospital pharmacy. We hypothesize that the items derived from the study by Malik et al. (5) can be effectively used to construct a reliable survey instrument. Additionally, we anticipate that the underlying factors will align with the qualitative findings of Malik et al. (5). Furthermore, we expect to find positive (*r* > 0.40) between the factors of OOC and the score on the Interpersonal Communication Inventory (ICI) (7).

## Method and analyses

### Study context and participants

At pharmacy departments of the university hospital, we aimed to train all healthcare personnel in basic mediation and professional communication skills. To evaluate the effectiveness of the training, participants completed the same questionnaire before and after the training sessions. The data or this study were derived from these trainings. All healthcare personnel from the two pharmacy departments at Erasmus MC – the in-patient and out-patient pharmacies – were invited to participate. The two pharmacies had comparable team compositions, backgrounds, education levels and job roles. There were no exclusion criteria; all employees were eligible to join. The effectiveness of the intervention was assessed using a before-and-after study design.

### Intervention

We designed a concise training program to equip participants with basic mediation skills, aimed at de-escalating tension in professional communication at an early stage (14). These classroom sessions also focused on fostering an open atmosphere to discuss differences among colleagues before conflicts escalate. The training included techniques derived from professional mediator training to maintain constructive dialogue during escalation or disagreement. Each team was asked to have 3 to 4 colleagues participate in three additional in-depth training sessions. All training sessions were conducted in a classroom setting and lasted 90 min each. This intervention took place between June 2022 and January 2023.

### Data collection

The questionnaires were distributed to the healthcare professionals’ hospital e-mails addresses using pre-programmed surveys in Castor EDC (version 2023.4.5.0), a web-based system designed for secure and valid data collection through electronic Case Report Forms (eCRFs). The system tracks completion rates and prevents duplicate or repeated entries. Participants received the questionnaires before and after the intervention period, referred to as the test phase and validation phase, respectively. The questionnaire measured various dimensions, including baseline characteristics (e.g., sex, years of work experience), the ICI, and 37 items assessing the self-perceived OOC within a healthcare department. The inclusion of OOC in both pre- and post-training questionnaires aimed to develop a reliable OOC survey instrument from these data.

After the intervention period, the same questionnaire was sent again, including the OOC items, regardless of intervention completion, to assess test/re-test reliability. The study was approved by the Medical Ethics Review Board of Erasmus MC (MEC-2022-0159), and written informed consent was obtained from all participants prior to participation. This study is part of a larger pre-registered project available on the Open Science Framework (available at: DOI 10.17605/OSF.IO/N8GE7). The COnsensus-based Standards for the selection of health status Measurement INstruments (COSMIN) checklist for patient-reported outcome measurement instruments is provided in the Supplementary material (15).

### Item development

The original English and Dutch statements were developed using the Delphi method (5). Two authors verified the content (i.e., MMH, MvD) in consultation with an English language editor at Erasmus MC. Subsequently, two authors (i.e., MMH and WJRR) reviewed the items to ensure they were understandable, clear, and unambiguous. Both the Dutch and English versions are available in Supplementary material A. These statements reflect healthcare workers’ perceptions of important aspects of an OOC, making them suitable for use as a survey instrument. In the original study, leadership, employee attributes, organizational processes, and, to some extent, patient orientation were identified as the main themes. Our approach allows us to confirm whether these themes also emerge when quantitatively measuring OOC in a department. While the original study identified the important dimensions and statements of OOC, it did not provide response categories for use as a survey instrument. For each statement, respondents rated their agreement on a 7-point Likert scale, ranging from [1] “completely agree” to [7] “completely disagree.” This scale was chosen to assess self-perceived OOC because it is well-suited measuring perceptions on a continuous scale ad avoids strong ceiling or floor effects (16, 17).

As the questionnaire was similar before and after the training, we expected the factor structure (i.e., which items load on which factors) to remain consistent before and after the training. However, we did expect that the levels of self-perceived OOC and associations may have changed due to the training. In other words, if a factor reflects a specific phenomenon before the training and the measure is internally consistent, it should continue to measure the same phenomenon. The factor structure should remain stable, though the levels of the measures may vary.

### Construct validity

The ICI, developed by Bienvenu (7), was included to assess its correlations with the OOC factors identified in the initial analysis. The ICI measures an individual’s ability to communicate effectively and listen well. We expected positive correlations (*r* > 0.40) between OOC factors and the ICI in the test phase.

### Statistical analysis

We analyze the data in three steps. First, we described the characteristics of the study respondents using descriptive statistics. Categorical variables are reported as number and frequencies. Second, during the test phase, we conducted an Exploratory Factor Analysis (EFA) to identify emerging factors, following established guidelines (18). A factor was retained if it had at least three statements loading onto it, with factor loadings of 0.50 or higher. Statements we retained and assigned to the corresponding factor also if they loaded high on one factor and relatively low on others (19). If these conditions were no met, the statement was removed and the EFA repeated. We also inspected statements for meaningful content in relation to the identified factors to ensure content validity and coherence. The Kaiser-Meyer-Olkin measure of sampling adequacy (KMO) and Bartlett’s test statistic (20, 21) were reported to assess the suitability of the data for factor analysis. A KMO value above 0.8 was considered adequate, and a Bartlett’s test *p*-value below 0.05 was sufficient to proceed with EFA. This process allowed us to present the final statement set with adequate factor loadings and qualitative label the factors. We also reported Cronbach’s alpha to reflect internal consistency. Second, we calculated Pearson correlation coefficients between the latent factors identified and examined correlations between the OOC dimensions and the ICI. Third, during the validation phase, we repeated the EFA, calculated correlations, created Bland–Altman plots, and assessed Cronbach’s alpha. Data management was performed using Castor EDC (version 2023.4.5.0) and R studio (version 4.2.1), while statistical analyses were conducted using SPSS 28.0.1.0. A *p*-value below 0.05 was considered statistically significant.

## Results

### Background characteristics

The background characteristics of the respondents in the test phase sample are presented in Table 1. The sample included 191 pharmacy employees, with a response rate of 39.7%. The majority were female (*n* = 161, 84%), most were between 20 and 40 years old (*n* = 114, 60%), and most worked day shifts only (*n* = 170, 89%).

**Table 1:** Respondent’s background characteristics (*n* = 191).

Total sample		191 (100)
Sex	Male	30 (16)
	Female	161 (84)
Age	20–40 years	114 (60)
	41–60 years	67 (35)
	>61 years	10 (5)
Department	Inpatient pharmacy	123 (64)
	Outpatient pharmacy	68 (36)
Shift work	Day shifts	170 (89)
	Switch day/night shifts	21 (11)
Tenure at department	<10 years	160 (84)
	11 to 20 years	16 (8)
	21 to 30 years	12 (6)
	31 to 40 years	3 (2)
Employment contract	Full-time	99 (52)
	Part-time	84 (44)
	Flexible-contract/Freelance	8 (4)

All variables are categorical and presented as number (with percentage in parentheses).

### Test phase

We conducted three EFAs to align the underlying data structure with content-meaningful factors. In the first EFA, we included all 37 items derived from the previous study (5). The analysis showed a KMO value pf 0.956 and a statistically significant Barlett’s test (Bartlett’s statistics = 6160.01, *p* < 0.001). Four factors were identified (eigenvalue>1.0), but 12 items that did not load significantly onto any factor (factor loadings <0.5), resulting in an explained variance of 62.6%. These 12 items were excluded in the second EFA.

In the second EFA, the KMO value remained at 0.956, and Barlett’s test was again statistically significant (Bartlett’s statistics = 3853.07, *p* < 0.001). This analysis revealed three factors (eigenvalue >1.0) with an explained variance of 64.2%. One item did not load significantly on any factor, and another loaded relatively well onto all three factors. Both items were deleted for the third EFA. Content validation showed that the remaining items were well-clustered around their respective factors.

The third and final EFA had a KMO value of 0.954 and significant Bartlett’s test (Bartlett’s statistics = 3453.30, *p* < 0.001). All retained items loaded strongly onto one factor and weakly on the others. We also confirmed that the items aligned with their respective factors based on content validity, which was the case. As a result, we included 22 items in the final set of questions, retaining three factors that explained 64.9% of the variance. Table 2 presents the original 37 items (in order presented to the respondents) and indicated which items were included in the final item set. The three factors were labelled: enabling systems, open behavior, and trusting and supporting coworkers. These factors and their associated items make sense from both content validity and theoretical perspectives, with items clustering logically under their respective factors. Supplementary material B provides the rotation matrices for all three EFAs and the results of each step.

**Table 2:** Original and retained items.

Item	English statement	Factor	Factor	Factor	Included?
		Enabling systems	Open behavior	Trusting and supporting coworkers	
1	We show interest in each other’s competences				Excluded
2	Our procedures and systems ensure transparency with regard to successes and points of improvement	0.621	0.273	0.262	Included
3	There is informal contact that strengthens cohesion within the team				Excluded
4	Our management or supervisor is well informed about the daily working routine and can take the right decisions	0.761	0.229	0.194	Included
5	We have faith in each other’s competencies	0.463	0.200	0.635	Included
6	We continuously improve based on what we have learnt from the feedback systems of our department	0.605	0.305	0.394	Included
7	Our management or supervisor helps us to solve problems	0.749	0.344	0.239	Included
8	We listen to each other’s opinions regardless of the hierarchy and take decisions on substantive grounds				Excluded
9	The patient structurally gives us feedback on the experienced care				Excluded
10	We do not blame each other for incidents	0.117	0.196	0.643	Included
11	Respect for colleagues and patients is one of our most important values	0.187	0.231	0.804	Included
12	We trust each other’s intentions	0.297	0.293	0.741	Included
13	We support each other emotionally in our department	0.223	0.242	0.651	Included
14	We sincerely approach each other positively, give each other compliments and express appreciation	0.484	0.338	0.606	Included
15	Colleagues with prestige also dare to be vulnerable				Excluded
16	Joint reflection on our actions and processes is structurally embedded in our work	0.581	0.317	0.349	Included
17	We invest in a learning environment in which people in training are allowed to challenge their supervisors				Excluded
18	We can indicate that we cannot cope with the high workload and if so, serious attention is being paid	0.659	0.413	0.253	Included
19	The views of the patients influences our policy				Excluded
20	We discuss in our department how we can prevent incidents from reoccurring				Excluded
21	We do not abuse power	0.283	0.594	0.400	Included
22	We feel free to question the decisions or actions of colleagues with authority	0.467	0.540	0.382	Included
23	We feel safe to be ourselves within the organization	0.321	0.651	0.438	Included
24	We feel comfortable in discussions to speak our minds when our thoughts deviate from the norm	0.318	0.800	0.292	Included
25	We are informed and involved with regard to changes in our department				Excluded
26	We can express constructive criticism without fear of negative consequences	0.361	0.785	0.233	Included
27	The culture in our department makes it easy to acknowledge mistakes and to learn from each other’s mistakes				Excluded
28	Our management or supervisor show exemplary behavior that fits into an open culture	0.674	0.487	0.300	Included
29	We dare to be open about our individual points of improvement and how they can be further developed	0.323	0.597	0.339	Included
30	Possible dysfunction is addressed in time and is constructively resolved				Excluded
31	We are aware of each other’s qualities and make sufficient use of them				Excluded
32	We are outspoken to each other and not about one another; should this be otherwise, we will call each other to account				Excluded
33	Difficult topics that stand in the way of openness, such as shame, fear, power, distrust and dysfunction, can be discussed openly				Excluded
34	We are open to views from a wide network, such as those of other departments, professions and institutions				Excluded
35	We recognize, value and stimulate diversity	0.307	0.512	0.395	Included
36	We experience low barriers to discuss ideas and issues with our management or supervisor	0.493	0.581	0.125	Included
37	We can listen to and watch others without judging immediately	0.415	0.382	0.536	Included

The rotated factor matrix of the final EFA is presented in the table. Numbers are factor loadings to the respective factor.

Table 3 presents the Cronbach’s alpha values and the correlations among the three factors. All three factors demonstrated a Cronbach’s alpha higher than 0.9. Additionally, the Pearson correlation coefficients showed strong correlations among the three factors (*r* > 0.7, *p* < 0.001). Significant positive correlations were also observed between the ICI and the three OOC factors: enabling systems (*r* = 0.37, *p* < 0.001), open behavior (*r* = 0.43, *p* < 0.001), and trusting and supporting coworkers (*r* = 0.47, *p* < 0.001). Supplementary material C provides the distributions of the scores, along with skewness and kurtosis of the three factors.

**Table 3:** Correlation matrix and Cronbach’s alpha.

			Factor		
		Mean (SD)	Enabling systems	Open behavior	Trusting and supporting coworkers
Factor	Enabling systems (*n* = 7 items)	3.51 (1.35)	1.00		
	Open behavior (*n* = 8 items)	3.00 (1.26)	0.81	1.00	
	trusting and supporting coworkers (*n* = 7 items)	2.79 (1.14)	0.72	0.74	1.00
	Interpersonal communication inventory	2.39 (0.30)	0.37	0.43	0.47
	Cronbach’s alpha		0.921	0.931	0.912

All Pearson’s correlations are significant with a *p* < 0.001. Sample size is 191 participants.

### Validation phase

**Figure 1:**
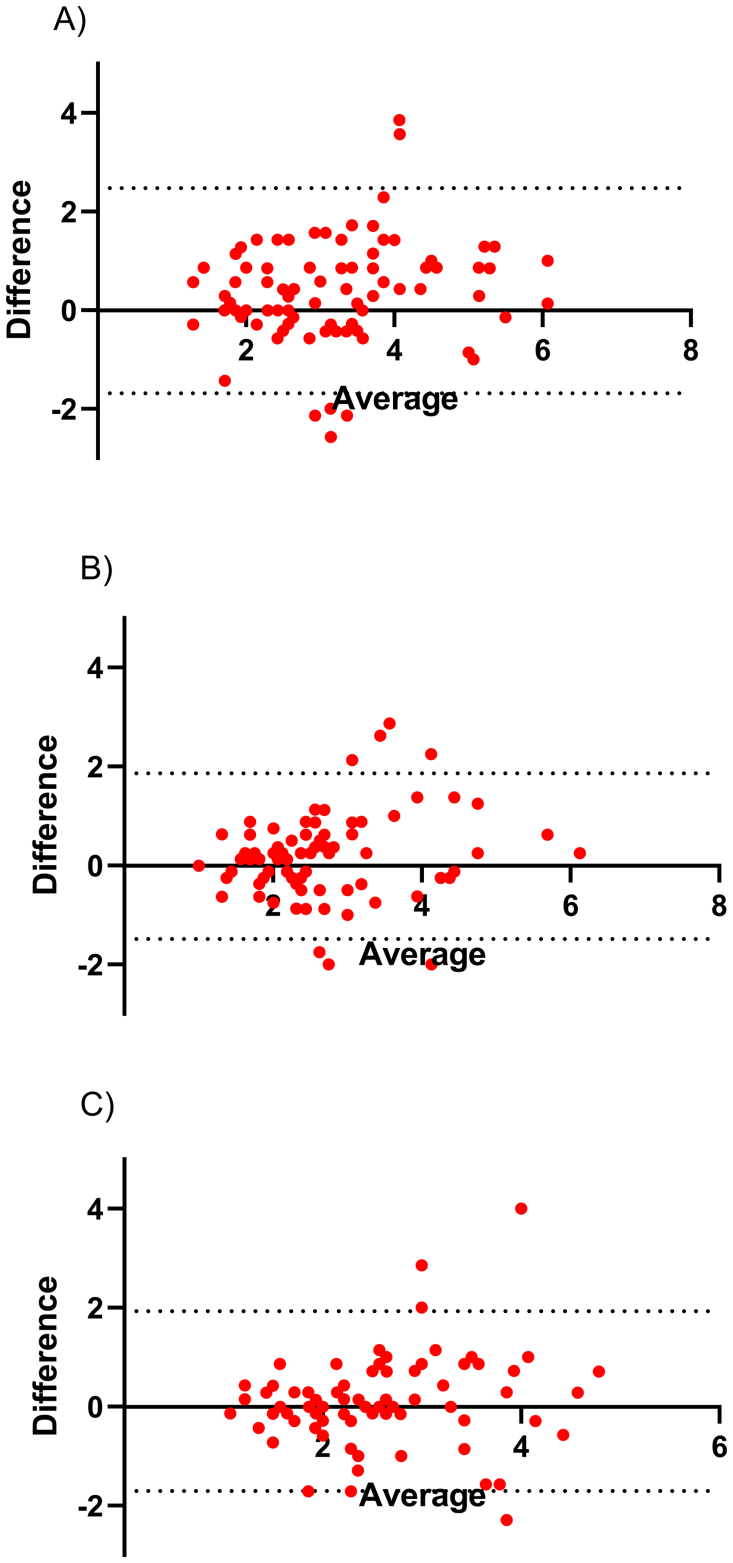
Bland–Altman plots. The Bland–Altman plots present the agreement between the identified factors in the test and validation-phase for **(A)** enabling systems, **(B)** open behavior, and **(C)** trusting and supporting coworkers. All factors show that there is a high degree of agreement. Taken together the Bland–Altman and high test–retest correlation this indicates that these factors are relatively reliable measures.

Approximately eight months after the intervention and initial survey, respondents were asked to complete the same questionnaire again. Of the 191 original respondents, 81 participated in the follow-up. We repeated the EFA using the 22 items identified in the test phase, constraining the solution to three factors (see Supplementary material D). The KMO statistic was 0.894, and Barlett’s test of sphericity was statistically significant (Bartlett’s statistics = 1358.07, *p* < 0.001). The results showed some inconsistencies regarding which items loaded strongly or poorly onto the originally defined factors. However, as shown in Table 4, when we considered the factors as identified in the test phase, we observed high Cronbach’s alphas for the three subscales in the validation phase (*α* > 0.88). Additionally, test/re-test correlations were strong for the factors: enabling processes (*r* = 0.65, *p* < 0.001), open behavior (*r* = 0.70, *p* < 0.001), and trusting and supporting coworkers (*r* = 0.56, *p* < 0.001). Figure 1 displays Bland–Altman plots showing high agreement between the test phase and validation phase outcomes for the three factors. Supplementary material E provide a comparison between the total sample in the test phase (*n* = 191) and the 81 respondents in the validation phase. No significant differences were found in the distributions of these variables, indicating no bias in loss to follow up.

**Table 4:** Validation-phase.

		Factor		
		Enabling systems	Open behavior	Trusting and supporting coworkers
Factor	Enabling systems	1.00		
	Open behavior	0.78	1.00	
	Trusting and supporting coworkers	0.63	0.73	1.00
	Cronbach’s alpha	0.882	0.915	0.891

All correlations are significant with a *p* < 0.001. Sample size is 81 participants.

## Discussion

This study aimed to construct a survey instrument to measure self-perceived OOC within university hospital pharmacies. An OOC is characterized by an environment where information, ideas, and feedback are freely exchanged among all members, regardless of their position and rank (4–6). From the original 37 statements, 22 items were selected to reflect three interrelated factors of an OOC in a university hospital pharmacy: enabling systems, open behavior, and trusting and supporting coworkers. The results indicated that these three factors were sufficient to explain a significant portion of the variance.

Of the original 37 items, 15 were excluded for various reasons. For example, the specific context of the pharmacy in this study may have led to the exclusion of certain patient-related items, such as item 9 (“the patient gives us feedback on the experienced care”), as pharmacists typically do not have direct contact with patients, despite their role in the care process. This item, however, may be more relevant in inpatient and outpatient clinical departments. Another excluded item, item 34 (“We are open to views from a wide network, such as those of other departments, professions and institutions”), might reflect an important aspect of an OOC but may be less perceptible within the pharmacy context.

Our study identified three factors associated with measuring self-perceived OOC in a healthcare department. These factors showed some overlap with those identified in the Delphi study by Malik et al. (5) (p. 8). The *first factor*, “enabling systems,” was measured by seven items, including: “Our procedures and systems ensure transparency with regard to successes and points of improvements” (item 2). Transparency about processes and improvements is crucial for fostering an OOC. Another item, “Management should lead by example and demonstrate behavior consistent with an OOC (item 28), emphasizes the importance of leadership in promoting openness. The items in this factor relate to how systems should facilitate an OOC within the department, which is why we labelled it “enabling systems.”

Organizations use both formal and informal methods to organize processes. By structuring processes to promote an OOC, they can facilitate professional communication among colleagues across hierarchies (5, 12) and foster fair, transparent decision-making (4). Additionally, an OOC may enhance work engagement and job satisfaction (22). Therefore, the factor “enabling systems” should focus on designing of a coherent set of processes that support and sustain an OOC.

The *second factor*, “open behavior,” was measured by 8 items. In an OOC, individual behavior significantly impacts the department as a whole. Each coworker should be mindful about their behavior within the department. This includes avoiding the abuse of power (item 21) and feeling comfortable expressing differing opinions, as reflected in item 24: “We feel comfortable in discussions to speak our minds when our thoughts deviate from the norm.” It also involves the ability to voice constructive criticism without fear of negative consequences, as captured in item 26: “we can express constructive criticism without fear of negative consequences.” Therefore, we labelled this factor “open behavior.”

In a department comprising diverse colleagues with varying personalities, individual open behavior is essential. Collectively, employees shape the department’s culture. As noted in previous research, on an individual level, showing interest and respect (5), and the ability to give and receive feedback (5) are key components in shaping an OOC. A critical element in this process is *professional socialization* (23), which refers to an individual’s journey to become familiar with the organization, department, processes, and culture. This is essential for existing and new employees to understand and actively participate in an OOC.

The *third factor*, “trusting and supporting coworkers,” was measured by 7 items. In an OOC, trust and support are crucial for effective collaboration among coworkers. The items associated with this factor emphasize these aspects. For example, “We have faith in each other’s competencies” (item 5), reflects the *trust* respondents have in their coworkers. Additionally, item 37 stated: “we can listen to and watch others without judging immediately.” This highlights the importance of trust and the ability to listen to each other without immediate judgement in fostering an OOC. Successful collaboration within a department requires trust and psychological safety (4, 5). Higher scores on these items indicate greater trust and support among coworkers, which, in turn, contributes to cultivating an OOC. Therefore, we labelled this third factor “trusting and supporting coworkers.”

To our knowledge, this is the first study to operationalize a measure of self-perceived OOC. Creating safe work environments is crucial, particularly in light of movements like “#me-too” movement, which have underscored the need for safety and openness in all workplaces, including healthcare (11). A safe environment allows individuals to express themselves freely about work-related aspects. Our survey instrument may help measure and monitor an OOC in a broader range of healthcare departments beyond just a pharmacy department.

The present study demonstrates that the self-perceived OOC measure has good test–retest reliability. The test–retest correlations, Cronbach’s alpha values, and Bland–Altman plots indicate that the 22 items and three factors are relatively stable over time. However, it remains possible that the intervention impacted the responses regarding self-perceived OOC, which future research should further explore.

Previous research has measured other aspects related to working in healthcare, such as safety attitudes towards patients, using the Safety Attitude Questionnaire (24), which assesses six domains related to a safety culture (e.g., teamwork and climate). This questionnaire originated in the Intensive Care Unit, focusing specifically on patient safety (24). While the concepts of patient safety and OOC are related, OOC takes a broader view of working in a healthcare setting. In addition to patient safety, healthcare workers should also focus on factors such as enabling systems fostering trust and support among colleagues. For example, this includes perceptions of how individuals function within the department and manage work load. We argue that fostering an OOC, where team members feel free to speak out and collaborate effectively, can also help safeguard patient safety.

## Limitations of the study and recommendations for future research

Some limitations discussed here should be considered for future studies. First, the response rate of 39.7% may be regarded as low for studies one of this nature. A possible reason for the low response rate could be that the research group is part of the same department, which may cause colleagues hesitant about participating in the training and survey.

Further, the training may have affected the level of self-perceived OOC and its associations with ICI. However, when the items are reflecting the same underlying factor and are internally consistent, we would expect a similar factor structure. Additionally, we did not correlate the emerging factors of self-perceived OOC and ICI during the validation phase. At this stage, following the classroom mediation skills intervention, perceptions of OOC in relation to ICI may have shifted. This could have influenced the strength of the correlations, which is why these analyses were not conducted in this study.

Also, because this study was conducted within the context of conflict resolution training, the findings may not be generalizable to broader context, and further validation is required. The fact that the researchers of the study were from the same department as the respondents may have also affected the responses due to potential relationships between them. Moreover, there was a significant loss to follow-up; of the original 191 respondents, only 81 respondents completed the survey after the training (Supplementary material E). This attrition may affect the results presented in the validation phase, and these findings should be interpreted with caution.

Future research should aim to confirm our findings using a confirmatory factor analysis, such as structural equation modeling. This approach would help further validate this instrument for research and management purposes. Our study provides an initial 22-item survey instrument to measure self-perceived OOC at large university hospital pharmacy. Further validation could be achieved by correlating scores on this scale with other personality and work-related factors. Consistent with our hypothesis, we found a significant positive correlation between the ICI and the dimensions of an OOC. Intuitively, an OOC is associated with high levels of ICI (7). Establishing construct validity is the next essential step in understanding self-perceived OOC (19).

To this end, the factors identified in our study should be correlated with other individual differences associated with an OOC and psychological safety at work. This approach may also be applied in other professional settings where an open OOC is considered beneficial for job performance. Future research may also correlate the OOC factors with established models, such as the Job-Demands Resources model (25), which explains factors influencing job performance. An OOC may reduce the “costs” associated with maintaining high job performance (i.e., job demands) while positively influencing employee “health” (i.e., resources). However, the specific effects of an OOC on the Job-Demand resources model require investigation in future studies.

## Conclusion

An OOC environment is characterized by free exchange of information, ideas, and feedback among all members, regardless of position or rank. To the best of our knowledge, no existing survey instrument specifically measures this type of culture within a healthcare department. This study aimed to develop a 22-item survey instrument to assess self-perceived OOC at a university hospital pharmacy. Our findings indicate that the survey instrument demonstrates internal consistency and shows evidence of construct validity. However, further validation and examination of its psychometric properties is recommended.

## Data Availability

The raw data supporting the conclusions of this article will be made available by the authors, without undue reservation.
