# Supplementary material for: Constructing a measure for self-perceived open organizational culture in a university hospital pharmacy
Source: Front Med (Lausanne). 2024 Oct 16;11:1428941. doi: 10.3389/fmed.2024.1428941 (PMC11521865; doi:10.3389/fmed.2024.1428941)
Supplement: Supplementary file 1 [file Data_Sheet_1.docx]

**SUPPLEMENTARY MATERIAL TO THE STUDY:**

**Constructing a measure for self-perceived open organizational culture in a university hospital pharmacy**

Wim J.R. Rietdijk^1,*^, Madzy Maljaars-Hendrikse^2,*^, Monique van Dijk^3^, Romana F. Malik^4^, Ngoc Tan^1^,

P. Hugo M. van der Kuy^1*^

^1^ Department of Clinical Pharmacy, Erasmus MC, Rotterdam, The Netherlands.

^2^ Legal Department, Erasmus MC, Rotterdam, The Netherlands.

^3^ Section Nursing Science, Department of Internal Medicine, Erasmus MC, Rotterdam, The Netherlands.

^4^ Department of Obstetrics and Gynaecology, Amsterdam University Medical Center, Amsterdam, the Netherlands

* Shared first authorship

Corresponding author: Wim Rietdijk PhD, Dr. Molewaterplein 40, 3015 GD, Rotterdam. E-mail: w.rietdijk@erasmusmc.nl.

**Keywords:** Open organizational culture, healthcare, validation, survey, reliability, construct validation.

**Table of Contents**

[COSMIN checklist for patient-reported outcome measurement instruments 3](#_Toc177979042)

[Supplementary Material A Items in the Dutch and English language 11](#_Toc177979043)

[Supplementary Material B Rotation matrix for three Exploratory Factor Analyses 14](#_Toc177979044)

[Supplementary Material C Distribution and skewness and kurtosis of the identified factors 17](#_Toc177979045)

[Supplementary Material D Rotation matrix validation EFA 18](#_Toc177979046)

[Supplementary Material E Comparing the total sample of 191 respondents in the test-phase with the remaining 81 respondents 19](#_Toc177979047)

# COSMIN checklist for patient-reported outcome measurement instruments

|  |  | Where in the study?* |
| --- | --- | --- |
| **GENERAL RECOMMENDATIONS** |  |  |
| *Research aim* | Provide clear research aim | Abstract; Introduction |
|  |  |  |
| *PROM* | Provide a clear research aim, including (1) the name and version of the PROM, (2) the target population, and (3) the measurement properties of interest | Abstract; Introduction |
|  |  |  |
|  | Provide a clear description of the construct to be measured | Introduction |
|  |  |  |
|  | Provide a clear description of the development process of the PROM, including a description of the target population for which the PROM was developed | Method and analyses |
|  |  |  |
|  | The origin of the construct should be clear: provide a theory, conceptual framework (i.e. reflective or formative model) or disease model used or clear rationale to define the construct to be measured | Introduction; Method and analyses |
|  |  |  |
|  | Provide a clear description of the structure of the PROM (i.e. the number of items and subscales included in the PROM, instructions given and response options) and its scoring algorithm | Method and analyses; Results; Discussion; Table 1; Supplementary Material Table A |
|  |  |  |
|  | Provide a clear description of existing evidence on the quality of the PROM | Introduction |
|  |  |  |
|  | Provide a clear description of the context of use | Introduction. Mainly to be used in a healthcare department setting. |
|  |  |  |
|  |  |  |
| *Target population* | Provide a clear description of in- and exclusion criteria to select patients, e.g. in terms of disease condition and characteristics like age, gender, language or country, and setting (e.g. general population, primary care or hospital/rehabilitation care) | Method and analyses |
|  |  |  |
|  | Provide a clear description of the method used to select the patients for the study (e.g. convenience, consecutive, or random) | Method and analyses |
|  |  |  |
|  | Describe whether the selected sample is representing the target population in which the PROM will be used in terms of age, gender, important disease characteristics (e.g. severity, status, duration) | The sample is representative for a hospital pharmacy. In line with other studies, the majority of the healthcare workers are women. Though, representativeness should be examined in future research. |
|  |  |  |
| **CONTENT VALIDITY** |  |  |
| *Design requirements* | From the perspective of professionals: use an appropriate method for assessing (1) the relevance of each item for the construct of interest, AND (2) the comprehensiveness of the PROM | The statement set was developed in a previous study. In this delphi method study, healthcare workers reflected on the meaning and content of an open culture. In our view, these are representative to use to study an open culture in healthcare departments. |
|  |  |  |
|  | Include professionals from all relevant disciplines | We have included healthcare workers from pharmacy departments. |
|  |  |  |
|  | Evaluate each item in an appropriate number of patients or professionals For qualitative studies For quantitative (survey) studies | The items were taken from a Delphi-method study. |
|  |  |  |
|  | Use skilled group moderators or interviewers | This was done in the previous study including a Delphi-method. |
|  |  |  |
|  | Base the group meetings or interviews on an appropriate topic or interview guide | This was done in the previous study including a Delphi-method. |
|  |  |  |
|  | Record and transcribe verbatim the group meetings or interviews | This was done in the previous study including a Delphi-method. |
|  |  |  |
|  | Use an appropriate approach to analyze the data | This was done in the previous study including a Delphi-method. |
|  |  |  |
|  | Involve at least two researchers in the analysis | This was done in the previous study including a Delphi-method. |
|  |  |  |
| **INTERNAL CONSISTENCY** |  |  |
|  | Check whether a scale or a subscale is unidimensional | Method and analyses; Results. |
|  |  |  |
|  | Perform the analysis in a sample with an appropriate number of patients (taking into account expected number of missing values) | We included 191 respondents, having a response rate of 39.4%. |
|  |  |  |
|  | Provide a clear description of how missing items will be handled | Method and analyses. |
|  |  |  |
|  | For continuous scores: calculate Cronbach’s alpha or Omega for each unidimensional scale or subscale | We calculated the Cronbach's alpha. Method and analysis; Results; Table 3; Table 4. |
|  |  |  |
|  | For dichotomous scores: calculate Cronbach’s alpha or KR-20 for each unidimensional scale or subscale | Not applicable. |
|  |  |  |
|  | For IRT-based scores: calculate standard error of theta (SE (θ)) or reliability coefficient of estimated latent trait value (index of (subject or item) separation) for each unidimensional scale or subscale | Not applicable. |
|  |  |  |
| **CONSTRUCT VALIDITY** |  |  |
|  | Formulate hypotheses about expected relationships between the PROM under study and other outcome measurement instrument(s) | Introduction; Method and analyses. |
|  |  |  |
|  | Provide a clear description of the construct(s) measured by the comparator instrument(s) | Method and analyses. |
|  |  |  |
|  | Use comparator instrument(s) with sufficient measurement properties | Method and analyses. |
|  |  |  |
|  | Perform the analysis in a sample with an appropriate number of patients (taking into account expected number of missing values) | Method and analyses; Results. |
|  |  |  |
|  | Use an appropriate time schedule for assessments of the PROM of interest and comparison instruments | Method and analyses; Results. |
|  |  |  |
|  | Use statistical methods that are appropriate for the hypotheses to be tested | Method and analyses; Results. |
|  |  |  |
|  | Provide a clear description of how missing items will be handled | Method and analyses; Results. |
|  |  |  |
| **TRANSLATION PROCESS** |  |  |
|  | Describe both the original language in which the PROM was developed, the source language (if different from the original language) and the language in which the PROM will be translated | Originally the statement set was collected in the Dutch language. They were translated in English for publication in the original study by Malik and colleagues. In personal contact we obtained both the English (published in the study) and the Dutch versions of the statements. |
|  |  |  |
|  | Ensure that the items will be translated forward and backward | See the Malik et al (2021) study. |
|  |  |  |
|  | Ensure that both forward translators have a mother tongue in the target language in which the PROM will be translated | See the Malik et al (2021) study. |
|  |  |  |
|  | Ensure that one of the forward translators has expertise in the diseases involved, and in the construct measured by the PROM; the other forward translators is naïve on the construct measured by the PROM | See the Malik et al (2021) study. |
|  |  |  |
|  | Ensure that both backward translators have a mother tongue in the original or source language | See the Malik et al (2021) study. |
|  |  |  |
|  | Ensure that both backward translators are naïve in the disease involved and the construct to be measured | See the Malik et al (2021) study. |
|  |  |  |
|  | Ensure that the translators will work independently from each other | See the Malik et al (2021) study. |
|  |  |  |
|  | Provide a clear description on how differences between the original and translated versions will be resolved | See the Malik et al (2021) study. |
|  |  |  |
|  | Ensure that the translation will be reviewed by a committee (including the original developers of the PROM) | See the Malik et al (2021) study. |
|  |  |  |
|  | Write a feedback report of the translation process | See the Malik et al (2021) study. |
|  |  |  |
|  | Perform a pilot study (e.g. cognitive interview study) to check (1) the relevance of each item for the patients’ experience with the condition, AND (2) the comprehensiveness of the PROM, AND (3) the comprehensibility of the PROM instructions, items, response options, and recall period | See the Malik et al (2021) study. |
|  |  |  |
|  | Perform the pilot study in a patient population representing the target population | See the Malik et al (2021) study. |
|  |  |  |
| **MEASUREMENT ERROR AND RELIABILITY** |  |  |
|  | Use at least two measurements | Method and analyses |
|  |  |  |
|  | Ensure that the administrations will be independent | Method and analyses |
|  |  |  |
|  | Ensure that the patients will be stable in the interim period on the construct to be measured | Method and analyses |
|  |  |  |
|  | Use an appropriate time interval between the two measurements, which is long enough to prevent recall, and short enough to ensure that patients remain stable | Method and analyses |
|  |  |  |
|  | Ensure that the test conditions will be similar for the measurements (e.g. type of administration, environment, instructions) | Method and analyses |
|  |  |  |
|  | Perform the analysis in a sample with an appropriate number of patients (taking into account expected number of missing values) | Method and analyses |
| *We provided the main sections where these checklist items are described. Where necessary, we provide more details in the table above. | | |

# Supplementary Material A Items in the Dutch and English language

| **Item** | **Statement in Dutch** | **Statement in English** | **Included?** |
| --- | --- | --- | --- |
| 1 | We tonen belangstelling voor elkaars competenties. | We show interest in each other’s competences | excluded |
| 2 | Onze procedures en systemen zorgen voor transparantie wat betreft successen en verbeterpunten. | Our procedures and systems ensure transparency with regard to successes and points of improvement | Included |
| 3 | Er is informeel contact dat de samenhang binnen het team versterkt. | There is informal contact that strengthens cohesion within the team | excluded |
| 4 | Onze leidinggevende is goed op de hoogte van het dagelijkse werk en kan de juiste beslissingen nemen. | Our management or supervisor is well informed about the daily working routine and can take the right decisions | Included |
| 5 | We hebben vertrouwen in elkaars competenties. | We have faith in each other’s competences | Included |
| 6 | We verbeteren onszelf en onze werkprocessen continu naar aanleiding van wat wij hebben geleerd van de feedbacksystemen van onze afdeling. | We continuously improve based on what we have learnt from the feedback systems of our department | Included |
| 7 | Ons management helpt ons om problemen op te lossen. | Our management or supervisor helps us to solve problems | Included |
| 8 | We luisteren naar elkaars mening ongeacht de hiërarchie en nemen beslissingen op inhoudelijke gronden. | We listen to each other’s opinions regardless of the hierarchy and take decisions on substantive grounds | excluded |
| 9 | De patiënt geeft ons structureel feedback op de ervaren zorg. | The patient structurally gives us feedback on the experienced care | excluded |
| 10 | We geven elkaar niet de schuld van incidenten. | We don't blame each other for incidents | Included |
| 11 | Respect voor collega's en patiënten is een van onze belangrijkste waarden. | Respect for colleagues and patients is one of our most important values | Included |
| 12 | We vertrouwen elkaars goede bedoelingen. | We trust each other’s intentions | Included |
| 13 | We steunen elkaar emotioneel op onze afdeling. | We support each other emotionally in our department | Included |
| 14 | We benaderen elkaar oprecht positief, geven elkaar complimenten en spreken waardering uit. | We sincerely approach each other positively, give each other compliments and express appreciation | Included |
| 15 | Collega's met aanzien durven zich ook kwetsbaar op te stellen. | Colleagues with prestige also dare to be vulnerable | excluded |
| 16 | Gezamenlijke reflectie op ons handelen en onze processen is structureel verankerd in ons werk. | Joint reflection on our actions and processes is structurally embedded in our work | Included |
| 17 | We investeren in een leeromgeving waarin mensen in opleiding hun begeleiders mogen uitdagen. | We invest in a learning environment in which people in training are allowed to challenge their supervisors | excluded |
| 18 | We kunnen het aangeven als we de hoge werkdruk niet aankunnen en dan wordt er serieus aandacht aan besteed. | We can indicate that we cannot cope with the high workload and if so, serious attention is being paid | Included |
| 19 | De mening van de patiënten beïnvloedt ons beleid. | The views of the patients influences our policy | excluded |
| 20 | We bespreken op onze afdeling hoe we kunnen voorkomen dat incidenten zich opnieuw voordoen. | We discuss in our department how we can prevent incidents from reoccurring | excluded |
| 21 | We maken geen misbruik van macht. | We do not abuse power | Included |
| 22 | We voelen ons vrij om beslissingen of handelingen van collega's met gezag in twijfel te trekken. | We feel free to question the decisions or actions of colleagues with authority | Included |
| 23 | We kunnen gerust onszelf zijn binnen de organisatie. | We feel safe to be ourselves within the organisation | Included |
| 24 | We voelen ons veilig een afwijkende mening te uiten in een discussie. | We feel comfortable in discussions to speak our minds when our thoughts deviate from the norm | Included |
| 25 | We worden geïnformeerd over en betrokken bij veranderingen op onze afdeling. | We are informed and involved with regard to changes in our department | excluded |
| 26 | We kunnen constructieve kritiek uiten zonder bang te zijn voor negatieve gevolgen. | We can express constructive criticism without fear of negative consequences | Included |
| 27 | De cultuur op onze afdeling maakt het gemakkelijk om fouten te erkennen en van elkaars fouten te leren. | The culture in our department makes it easy to acknowledge mistakes and to learn from each other’s mistakes | excluded |
| 28 | Ons management of leidinggevenden vertonen voorbeeldgedrag dat past bij een open cultuur. | Our management or supervisor show exemplary behaviour that fits into an open culture | Included |
| 29 | We durven open te zijn over onze eigen verbeterpunten en hoe deze verder ontwikkeld kunnen worden. | We dare to be open about our individual points of improvement and how they can be further developed | Included |
| 30 | Mogelijk slecht functioneren wordt tijdig aangepakt en constructief opgelost. | Possible dysfunction is addressed in time and is constructively resolved | excluded |
| 31 | We kennen elkaars kwaliteiten en maken daar voldoende gebruik van. | We are aware of each other’s qualities and make sufficient use of them | excluded |
| 32 | We spreken met elkaar en niet over elkaar; Zo niet, dan spreken we elkaar hierop aan. | We are outspoken to each other and not about one another; should this be otherwise, we will call each other to account | excluded |
| 33 | Moeilijke onderwerpen die openheid in de weg staan, zoals schaamte, angst, macht, wantrouwen en disfunctioneren, kunnen openlijk worden besproken. | Difficult topics that stand in the way of openness, such as shame, fear, power, distrust and dysfunction, can be discussed openly | excluded |
| 34 | We staan open voor meningen van andere afdelingen, beroepen en instellingen. | We are open to views from a wide network, such as those of other departments, professions and institutions | excluded |
| 35 | We erkennen, waarderen en stimuleren diversiteit. | We recognize, value and stimulate diversity | Included |
| 36 | We ervaren dat ideeën en problemen laagdrempelig met onze leidinggevende en het afdelingshoofd kunnen worden besproken. | We experience low barriers to discuss ideas and issues with our management or supervisor | Included |
| 37 | We luisteren naar elkaar zonder meteen een oordeel te vellen | We can listen to and watch others without judging immediately | Included |

# Supplementary Material B Rotation matrix for three Exploratory Factor Analyses

***Below you find the rotation matrix of the first Exploratory Factor Analysis (EFA).***

| EFA | Factors | | | |  |  |
| --- | --- | --- | --- | --- | --- | --- |
| Item | 1 | 2 | 3 | 4 |  |  |
| 1 | 0.424 | 0.408 | 0.421 | 0.169 | excluded |  |
| 2 | 0.309 | 0.654 | 0.211 | 0.131 | included |  |
| 3 | 0.228 | 0.463 | 0.403 | 0.062 | excluded |  |
| 4 | 0.383 | 0.663 | 0.155 | 0.090 | included |  |
| 5 | 0.214 | 0.477 | 0.575 | 0.233 | included |  |
| 6 | 0.319 | 0.615 | 0.328 | 0.231 | included |  |
| 7 | 0.446 | 0.666 | 0.190 | 0.230 | included |  |
| 8 | 0.485 | 0.413 | 0.459 | 0.301 | excluded |  |
| 9 | -0.034 | 0.513 | 0.306 | 0.117 | excluded |  |
| 10 | 0.143 | 0.130 | 0.621 | 0.245 | included |  |
| 11 | 0.221 | 0.212 | 0.778 | 0.135 | included |  |
| 12 | 0.306 | 0.287 | 0.742 | 0.099 | included |  |
| 13 | 0.243 | 0.229 | 0.626 | 0.161 | included |  |
| 14 | 0.394 | 0.491 | 0.596 | 0.039 | included |  |
| 15 | 0.481 | 0.440 | 0.293 | 0.123 | excluded |  |
| 16 | 0.374 | 0.518 | 0.290 | 0.235 | included |  |
| 17 | 0.449 | 0.471 | 0.149 | 0.302 | excluded |  |
| 18 | 0.529 | 0.608 | 0.202 | 0.115 | included |  |
| 19 | 0.057 | 0.467 | 0.274 | 0.183 | excluded |  |
| 20 | 0.340 | 0.381 | 0.220 | 0.361 | excluded |  |
| 21 | 0.573 | 0.145 | 0.398 | 0.397 | included |  |
| 22 | 0.579 | 0.387 | 0.368 | 0.173 | included |  |
| 23 | 0.637 | 0.257 | 0.432 | 0.207 | included |  |
| 24 | 0.784 | 0.218 | 0.320 | 0.156 | included |  |
| 25 | 0.459 | 0.595 | 0.129 | 0.214 | excluded |  |
| 26 | 0.797 | 0.234 | 0.244 | 0.223 | included |  |
| 27 | 0.473 | 0.344 | 0.364 | 0.441 | excluded |  |
| 28 | 0.579 | 0.577 | 0.267 | 0.181 | included |  |
| 29 | 0.566 | 0.246 | 0.327 | 0.288 | included |  |
| 30 | 0.363 | 0.583 | 0.365 | 0.181 | excluded |  |
| 31 | 0.384 | 0.376 | 0.415 | 0.375 | excluded |  |
| 32 | 0.401 | 0.417 | 0.491 | 0.168 | excluded |  |
| 33 | 0.514 | 0.441 | 0.393 | 0.269 | included |  |
| 34 | 0.303 | 0.255 | 0.242 | 0.828 | included |  |
| 35 | 0.423 | 0.247 | 0.347 | 0.540 | included |  |
| 36 | 0.683 | 0.330 | 0.104 | 0.174 | included |  |
| 37 | 0.368 | 0.366 | 0.508 | 0.325 | included |  |
| Note: We used maximum likelihood varimax with kaiser normalization extraction method implemented in SPSS. We found four factors with an eigenvalue above 1 with a cumulative explained variance of 62.6%. Scree plot indicated around three factors. | | | | | |  |
|  |  |  |  |  |  |  |

***Below you find the rotation matrix of the second EFA. In this analysis, 12 items were dropped as compared to the first EFA (above).***

| Exploratory factor analysis - Second run | Factor | | |  |
| --- | --- | --- | --- | --- |
| retained items | 1 | 2 | 3 |  |
| 2 | 0.621 | 0.275 | 0.259 |  |
| 4 | 0.761 | 0.232 | 0.190 |  |
| 5 | 0.462 | 0.208 | 0.635 |  |
| 6 | 0.602 | 0.312 | 0.393 |  |
| 7 | 0.748 | 0.354 | 0.237 |  |
| 10 | 0.112 | 0.207 | 0.644 |  |
| 11 | 0.187 | 0.239 | 0.800 |  |
| 12 | 0.299 | 0.295 | 0.732 |  |
| 13 | 0.224 | 0.250 | 0.652 |  |
| 14 | 0.488 | 0.335 | 0.599 |  |
| 16 | 0.577 | 0.324 | 0.346 |  |
| 18 | 0.661 | 0.416 | 0.248 |  |
| 21 | 0.271 | 0.617 | 0.399 |  |
| 22 | 0.467 | 0.542 | 0.375 |  |
| 23 | 0.322 | 0.650 | 0.430 |  |
| 24 | 0.322 | 0.790 | 0.285 |  |
| 26 | 0.357 | 0.789 | 0.225 |  |
| 28 | 0.670 | 0.494 | 0.294 |  |
| 29 | 0.315 | 0.605 | 0.335 |  |
| 33 | 0.489 | 0.506 | 0.420 |  |
| 34 | 0.332 | 0.462 | 0.360 |  |
| 35 | 0.295 | 0.539 | 0.398 |  |
| 36 | 0.489 | 0.583 | 0.119 |  |
| 37 | 0.408 | 0.398 | 0.538 |  |
| Note: We used maximum likelihood varimax with kaiser normalization extraction method implemented in SPSS. We found three factors with an eigenvalue above 1 with a cumulative explained variance of 64.2%. Scree plot indicated three factors. | | | |  |
|  |  |  |  |  |
|  |  |  |  |  |
|  |  |  |  |  |

***Below you find the rotation matrix of the third EFA. In this analysis, two items were dropped as compared to the second EFA (above).***

| Exploratory factor analysis - Third run | Factor | | |  |
| --- | --- | --- | --- | --- |
| retained items | 1 | 2 | 3 |  |
| 2 | 0.621 | 0.273 | 0.262 |  |
| 4 | 0.761 | 0.229 | 0.194 |  |
| 5 | 0.463 | 0.200 | 0.635 |  |
| 6 | 0.605 | 0.305 | 0.394 |  |
| 7 | 0.749 | 0.344 | 0.239 |  |
| 10 | 0.117 | 0.196 | 0.643 |  |
| 11 | 0.187 | 0.231 | 0.804 |  |
| 12 | 0.297 | 0.293 | 0.741 |  |
| 13 | 0.223 | 0.242 | 0.651 |  |
| 14 | 0.484 | 0.338 | 0.606 |  |
| 16 | 0.581 | 0.317 | 0.349 |  |
| 18 | 0.659 | 0.413 | 0.253 |  |
| 21 | 0.283 | 0.594 | 0.400 |  |
| 22 | 0.467 | 0.540 | 0.382 |  |
| 23 | 0.321 | 0.651 | 0.438 |  |
| 24 | 0.318 | 0.800 | 0.292 |  |
| 26 | 0.361 | 0.785 | 0.233 |  |
| 28 | 0.674 | 0.487 | 0.300 |  |
| 29 | 0.323 | 0.597 | 0.339 |  |
| 35 | 0.307 | 0.512 | 0.395 |  |
| 36 | 0.493 | 0.581 | 0.125 |  |
| 37 | 0.415 | 0.382 | 0.536 |  |
| Note: We used maximum likelihood varimax with kaiser normalization extraction method implemented in SPSS. We found three factors with an eigenvalue above 1 with a cumulative explained variance of 64.9%. Scree plot indicated three factors. | | | |  |
|  |  |  |  |  |
|  |  |  |  |  |

# Supplementary Material C Distribution and skewness and kurtosis of the identified factors

These data present the distribution of scores of the respondents in the test-phase of the study on the Open Organizational Culture (OOC) factors: enabling systems, open behavior, and trusting and supporting coworkers.

Skewness: 0.476

Kurtosis: -0.645

Skewness: 0.937

Kurtosis: -0.355

Skewness: 0.953

Kurtosis: 1.020


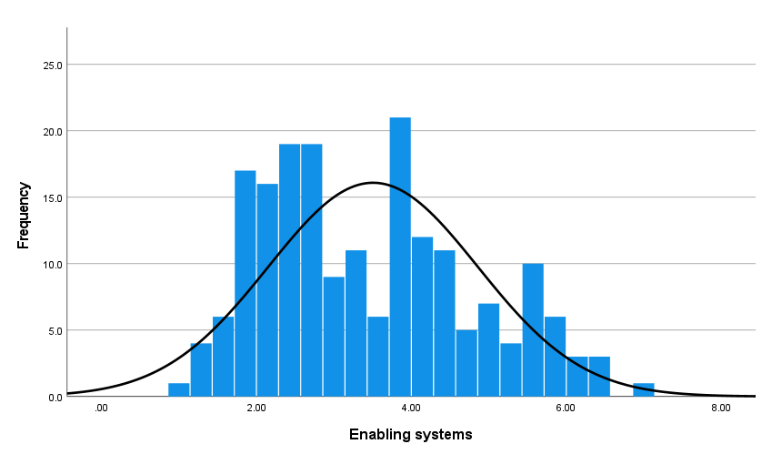

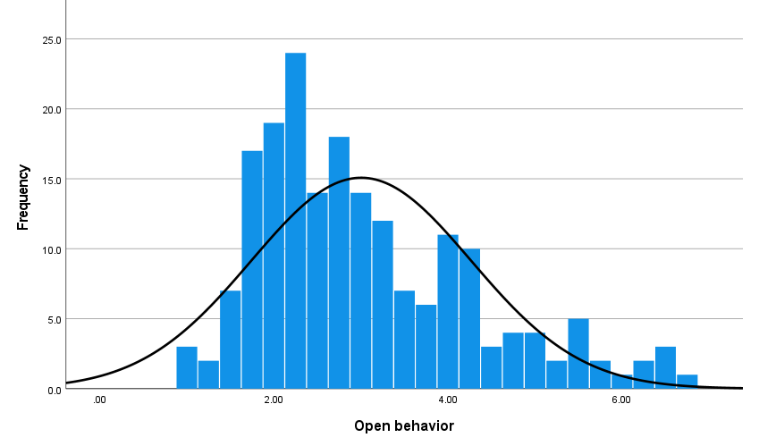

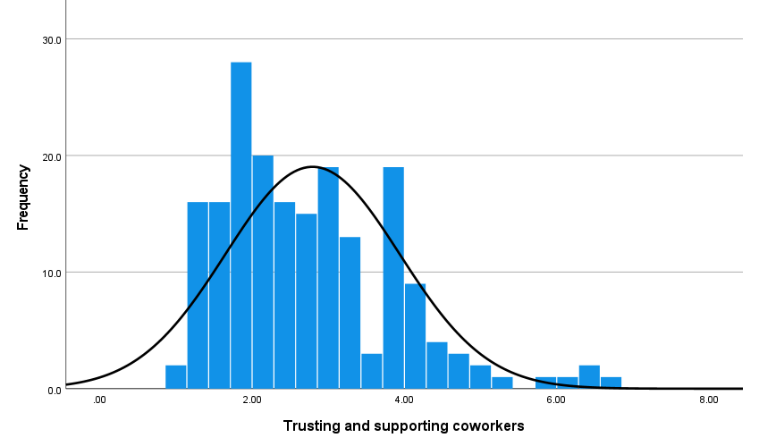


# Supplementary Material D Rotation matrix validation EFA

| EFA - Validation phase | Factor | | |
| --- | --- | --- | --- |
| retained items | 1 | 2 | 3 |
| 2 | 0.497 | 0.268 | 0.086 |
| 4 | 0.874 | 0.129 | 0.214 |
| 5 | 0.578 | 0.560 | 0.221 |
| 6 | 0.391 | 0.438 | 0.204 |
| 7 | 0.803 | 0.209 | 0.271 |
| 10 | 0.093 | 0.510 | 0.361 |
| 11 | 0.210 | 0.831 | 0.174 |
| 12 | 0.292 | 0.686 | 0.277 |
| 13 | 0.181 | 0.586 | 0.193 |
| 14 | 0.151 | 0.770 | 0.199 |
| 16 | 0.340 | 0.348 | 0.275 |
| 18 | 0.667 | 0.277 | 0.345 |
| 21 | 0.336 | 0.325 | 0.679 |
| 22 | 0.349 | 0.216 | 0.645 |
| 23 | 0.216 | 0.493 | 0.742 |
| 24 | 0.354 | 0.396 | 0.666 |
| 26 | 0.531 | 0.366 | 0.483 |
| 28 | 0.747 | 0.070 | 0.459 |
| 29 | 0.401 | 0.260 | 0.438 |
| 35 | 0.146 | 0.458 | 0.418 |
| 36 | 0.487 | 0.237 | 0.585 |
| 37 | 0.228 | 0.604 | 0.405 |
| Note: We used maximum likelihood varimax with kaiser normalization extraction method implemented in SPSS. We forced three factors to the EFA as identified in the test phase. The cumulative explained variance of the three factor is 59.6% . | | | |

# Supplementary Material E Comparing the total sample of 191 respondents in the test-phase with the remaining 81 respondents

**In the validation-phase.**

|  |  | 191 (100) | 81 (100) |
| --- | --- | --- | --- |
| Gender | *Male* | 30 (16) | 12 (15) |
|  | *Female* | 161 (84) | 69 (85) |
| Age | 20 - 40 years | 114 (60) | 42 (52) |
|  | *41 - 60 years* | 67 (35) | 33(41) |
|  | *>61 years* | 10 (5) | 6 (7) |
| Department | *Hospital pharmacy* | 123 (64) | 54 (66) |
|  | *Outpatient pharmacy* | 68 (36) | 27 (33) |
| Shift work | *Day shifts* | 170 (89) | 71 (88) |
|  | *Switch day/night shifts* | 21 (11) | 19 (12) |
| Tenure at department | *<10 years* | 160 (84) | 63 (78) |
|  | *Between 11 - 20 years* | 16 (8) | 7 9) |
|  | *Between 21-30 years* | 12 (6) | 9 (11) |
|  | *Between 31-40 years* | 3 (2) | 2 (3) |
| Employment contract | *Full-time* | 99 (52) | 38 (47) |
|  | *Part-time* | 84 (44) | 41 (51) |
|  | *Flexible-contract/Freelance* | 8 (4) | 2 (3) |
| Note: All variables are categorical. Categorical variables are presented as number (with percentages in parentheses). | | | |
|  |  |  |  |
